# The relationship between education attainment and gout, and the mediating role of modifiable risk factors: a Mendelian randomization study

**DOI:** 10.3389/fpubh.2023.1269426

**Published:** 2024-01-08

**Authors:** Xin Huang, Xin Chen, Qixi Liu, Zhiwei Zhang, Juan Miao, Yuchan Lai, Jinqing Wu

**Affiliations:** ^1^Department of Orthopedics, Mindong Hospital Affiliated to Fujian Medical University, Fuan, Fujian Province, China; ^2^Department of Urology, Mindong Hospital Affiliated to Fujian Medical University, Fuan, Fujian Province, China; ^3^Department of Nursing, Mindong Hospital Affiliated to Fujian Medical University, Fuan, Fujian Province, China

**Keywords:** education attainment, gout, mediation, Mendelian randomization study, causal relationship

## Abstract

**Objective:**

To investigate the causal relationship between educational attainment (EA) and gout, as well as the potential mediating effects of individual physical status (IPS) such as body mass index (BMI) and systolic blood pressure (SBP) and lifestyle habits (LH) including alcohol intake frequency (drinking), current tobacco smoking (smoking), and time spent watching television (TV).

**Methods:**

Utilizing two-sample Mendelian randomization (MR), we analyzed the causal effects of EA on gout risk, and of IPS (BMI and SBP) and LH (smoking, drinking, and TV time) on gout risk. Multivariable MR (MVMR) was employed to explore and quantify the mediating effects of IPS and LH on the causal relationship between EA and gout risk.

**Results:**

An elevation of educational attainment by one standard deviation (4.2 years) exhibited a protective effect against gout (odds ratio 0.724, 95% confidence interval 0.552–0.950; *p* = 0.020). We did not observe a causal relationship between smoking and gout, but BMI, SBP, drinking, and TV time were found to be causal risk factors for gout. Moreover, BMI, SBP, drinking, and TV time acted as mediating factors in the causal relationship between EA and gout risk, explaining 27.17, 14.83, 51.33, and 1.10% of the causal effects, respectively.

**Conclusion:**

Our study indicates that having a genetically predicted higher level of EA may provide protection against gout. We found that this relationship is influenced by IPS factors such as BMI and SBP, as well as LH including drinking and TV time.

## 1 Introduction

Gout is a widespread inflammatory arthritis characterized by the accumulation of sodium urate crystals in joint and non-articular structures (1). Gout is a chronic condition characterized by intermittent attacks that can severely impact a patient’s joint mobility and overall quality of life (2). These attacks typically target the first metatarsophalangeal joint, resulting in fever, swelling, and excruciating pain that can persist for several days to weeks (3). If left uncontrolled, the condition may progress to involve other joints such as the elbows, wrists, and hands, further exacerbating the patient’s symptoms (4).

Emerging epidemiological evidence highlights the rising prevalence and incidence of gout, shedding light on pressing social issues (5). Gout is a condition that exhibits significant geographic variation in prevalence, with the highest rates observed in Oceania, particularly among indigenous and South Pacific island populations, where the prevalence can exceed 10 percent. In contrast, the prevalence of gout in Europe during the period of 2003–2014 ranged from 1 to 4 percent (6). The pathogenesis of gout is multifactorial, with hyperuricemia being the most significant risk factor for its development (7). Environmental factors, such as the consumption of purine-rich foods, can increase serum uric acid levels, thereby promoting the development of gout (1). Interestingly, previous explorations have shown a hypothetical association between decreased education attainment (EA) and an increased tendency to manifest gout (8–10). Behaviors and lifestyles play a crucial role in regulating the overall health of the human body (11). Factors such as obesity, high blood pressure, alcohol consumption, smoking, and sedentary behavior have been identified as potential associated factors for gout (7, 12–14). The aforementioned IPS (BMI, SBP) and LH (drinking, smoking, TV time) may act as mediators in the association between educational attainment and the onset of gout.

Mendelian randomization (MR) has emerged as a novel genetic epidemiological approach, leveraging genetic variants, such as single-nucleotide polymorphisms (SNPs), as instrumental variables (IVs) to estimate the impact of exposures (i.e., EA) on outcomes (i.e., Gout). The distinctive feature of MR lies in the random allocation of SNPs during pregnancy, unaffected by postnatal factors like lipid levels. Consequently, the biases induced by residual confounding are minimized, and it serves as a bulwark against the confounding and reverse causality issues that are often pervasive in conventional observational studies (15, 16).

Here, we investigated the impact of EA on gout through two-sample MR and investigated the role of IPS and LH in mediating the causal effect of EA on gout risk through multivariable MR (MVMR).

## 2 Methodology

### 2.1 Overall study design

The summary data utilized in the MR analyses were obtained from publicly available databases, which had already received ethical approval from the respective studies’ ethics committees (17–20). Hence, no additional ethical clearance was required for this MR analysis. The study employed a two-step, two-sample MR design to investigate the potential mediating role of IPS (BMI, SBP) and LH (smoking, drinking, and TV time) in the relationship between EA and gout risk. This study is conducted in three distinct phases. The first phase involves the determination of the causal impact of EA on gout, IPS, and LH. The second phase focuses on establishing the causal influence of IPS and LH on gout. In the third phase, we aim to investigate and quantify the potential mediating role of IPS and LH in the causal relationship between EA and gout.

### 2.2 Data sources

Education Levels (Self-Reported at Age ≥ 30) were analyzed using data from the Social Science Genetic Association Consortium (SSGAC) database, which includes 766,345 participants of European ancestry (17). The analysis encompassed 10,101,242 SNP data points related to the genetic makeup of individuals with European heritage (further details for each cohort are provided in the Supplementary materials). Each major educational qualification was aligned with the international standard classification of education to derive equivalent years of education. Specifically, one standard deviation represents an additional 4.2 years of education. Population-level data on gout was incorporated from the R5 version of the GWAS conducted by the FinnGen research project.^1^ This vast dataset involved 150,797 participants of European descent and comprised information on 16,380,152 SNPs. Among this population, there were 3,576 diagnosed cases of gout, with a control group consisting of 147,221 unaffected individuals.

As for GWAS data on potential mediator factors, relevant information was obtained from various consortia. BMI data were sourced from the Genetic Investigation of Anthropometric Traits consortium, encompassing 2,630,552 samples (18). Simultaneously, the International Consortium of Blood Pressure provided data on SBP for 757,601 samples in this study (19). Furthermore, the comprehensive data on current tobacco smoking and alcohol intake frequency were derived from the Medical Research Council Integrative Epidemiology Unit (MRC-IEU). This dataset comprises a substantial cohort of 462,434 and 462,346 individuals of European descent drawn from the UK Biobank, with 9,851,867 of these SNPs associated with current smoking and drinking frequency.^2^ The UK Biobank represents a monumental prospective cohort study, encompassing approximately half a million adults aged 40 to 69, hailing from 22 centers across the United Kingdom (20). Data concerning time spent watching television were collected by the Neale lab from a study containing 319,740 samples.^3^ All of the above participants were of European descent and there was no gender discrimination. The exposure data set and the outcome data set are from different databases, and there is no sample overlap. Table 1 gives specifics about the data sources.

**Table 1:** Details of datasets included in analyses.

	Trait	Sample size (Case/Control)	Population	Consortium	Reference	GWAS-ID	SNPs	Year
Exposures	Education attainment	766,345	European	SSGAC	James J. Lee et al.	ieu-a-1239	10,101,242	2018
Outcome	Gout	3,576/ 147,221	European	Finngen	https://www.finngen.fi/en	finn-b-M13_GOUT	16,380,152	2021
Mediators	BMI	152,893/ 2,477,659	European	GIANT	Adam E. Locke et al.	ieu-a-785	2,477,659	2015
	SBP	757,601	European	ICBP	Evangelos Evangelou et al.	ieu-b-38	7,088,083	2018
	Current tobacco smoking	462,434	European	MRC-IEU	Gibran Hemani et al.	ukb-b-223	9,851,867	2018
	Alcohol intake frequency	462,346	European	MRC-IEU	Gibran Hemani et al.	ukb-b-5779	9,851,867	2018
	Time spent watching television	319,740	European	Neale Lab	Neale et al.	ukb-a-5	10,894,596	2017

SSGAC, Social Science Genetic Association Consortium; BMI, body mass index; GIANT, Genetic Investigation of ANthropometric Traits; SBP, systolic blood pressure; ICBP, International Consortium of Blood Pressure; MRC-IEU, Medical Research Council Integrative Epidemiology Unit.

### 2.3 SNP selection and quality control

In genetic variation studies aimed at investigating causality, the careful selection of SNPs as IVs and rigorous quality control measures are crucial to ensure the reliability of the findings. The MR analysis in this study is based on three fundamental assumptions (21): (a) strong correlation between IVs and the exposure factor of interest, (b) independence of IVs from confounding factors, and (c) IVs affecting the outcome solely through the exposure. To meet these assumptions, a systematic approach for SNP selection and quality control is followed. Initially, a strict *p*-value threshold (*p* < 5 × 10^(−8)) and pairwise linkage disequilibrium (LD) parameters (R squared <0.001 and a clump window >10,000 kB between SNPs) are applied to include only SNPs significantly associated with the exposure variable (22). The strength of each SNP’s effect is assessed by calculating the F-statistic using the formula F = beta^(2)/se^(2), where “beta” represents the effect size of the SNP and “se” represents the corresponding standard error. SNPs with an F-statistic below 10 are excluded to eliminate weakly correlated instruments from further analysis (23). Furthermore, a rigorous filtering process is implemented to systematically exclude palindromic or incompatible SNPs, ensuring the validity and compatibility of the selected IVs. Additionally, to identify and remove any SNPs that may exhibit pleiotropic effects, the MR-Pleiotropy Residual Sum and Outlier (MR-PRESSO) test is employed (24). After this comprehensive screening process, a refined set of SNPs is retained and utilized as IVs in subsequent MR analyses.

### 2.4 Statistical analysis

A two-step MR approach, a novel method for causal mediation analysis, was employed to investigate the mediating effects of IPS (BMI, SBP) and LH (smoking, drinking, TV time) on the relationship between EA and gout risk. In the first step, the causal effects of education level on BMI, SBP, smoking, drinking, TV time, and gout were estimated through two-sample univariable MR analysis. For the bivariate MR analysis, the inverse-variance weighted (IVW) method with random effects was used as the primary statistical analysis. The IVW method combines the results from each SNP using a random-effects meta-analysis with the multiplication of SNP-specific effects, which is typically the most reliable when the total is valid (25). Supplementary validations were also conducted using MR Egger and weighted median methods (26, 27). The second step involved the utilization of the MVMR method to explore and quantify the potential mediating effects of IPS and LH on the causal relationship between EA and gout (28). Specifically, the estimate of the effect of EA on gout was multiplied by the estimate of the effect of IPS/LH on gout. The total causal effect of EA on gout was then divided by the mediating effect to obtain the IPS/LH-mediated ratio.

To ensure the validity and robustness of the results, various sensitivity analyses were conducted. The heterogeneity of the SNPs used was assessed by performing Cochrane’s Q test, with *p*-values below 0.05 considered indicative of noteworthy heterogeneity (29). Furthermore, the presence of horizontal pleiotropy was examined by analyzing the intercept obtained through MR Egger analysis, with *p*-values below 0.05 suggestive of horizontal pleiotropy (16).

**Figure 1:**
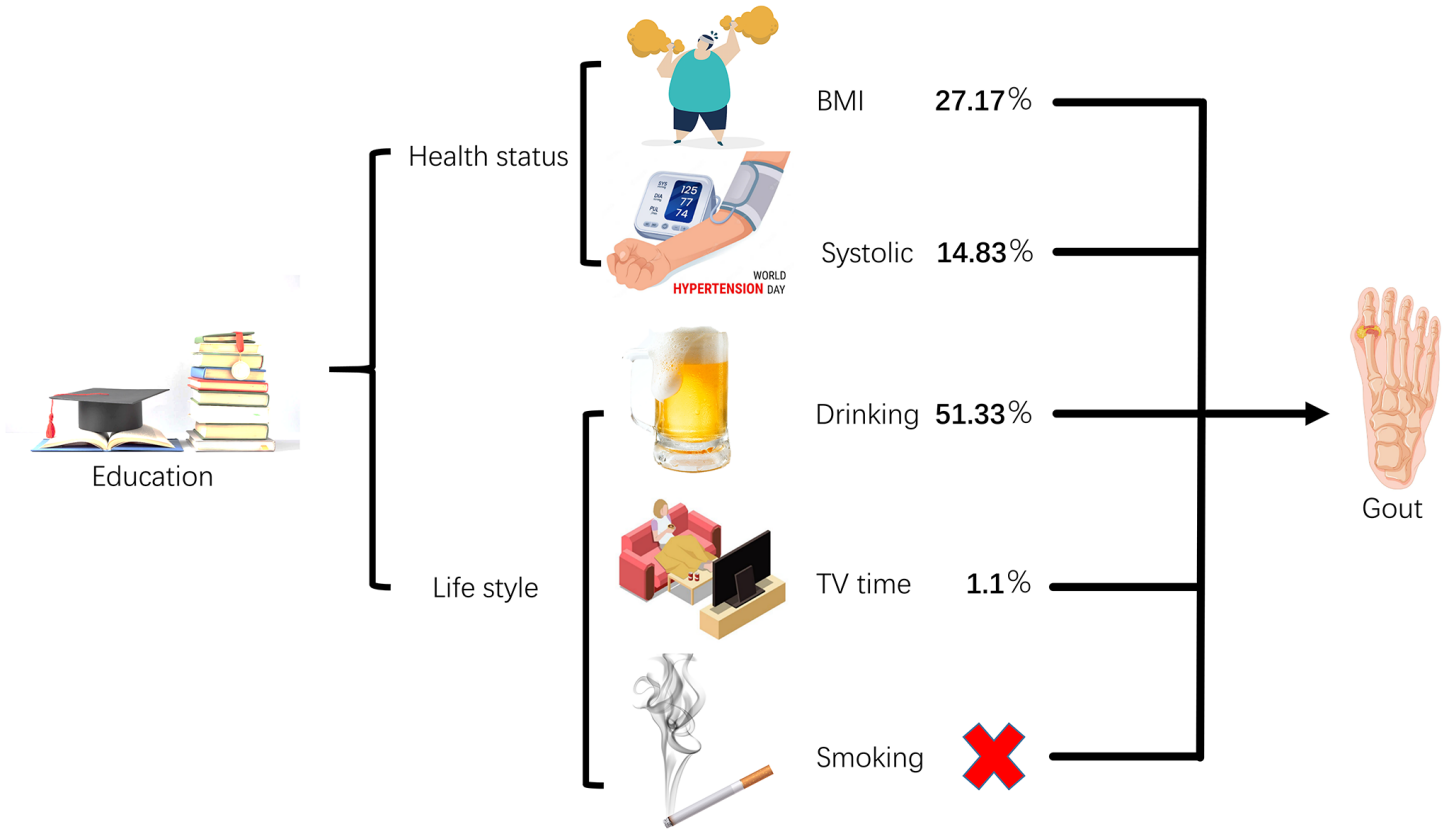
Overview of the study.

The MR analysis was performed using the TwoSampleMR package and MR-PRESSO in R version 4.2.0. The data and code for this study can be obtained from the corresponding author upon reasonable request (Figure 1).

## 3 Result

After rigorous exclusion of SNPs that did not meet strict quality control criteria (*p* < 5 × 10(−8), R2 < 0.001, *F* > 10), and subsequent removal of palindromic SNPs and incompatible SNPs, the remaining SNPs were subjected to MR analysis following the implementation of the MR-PRESSO method to mitigate potential outliers. Details of the SNPs can be found in the Supplementary material.

### 3.1 Overall causal effect of EA on gout, BMI, SBP, and LH

**Figure 2:**
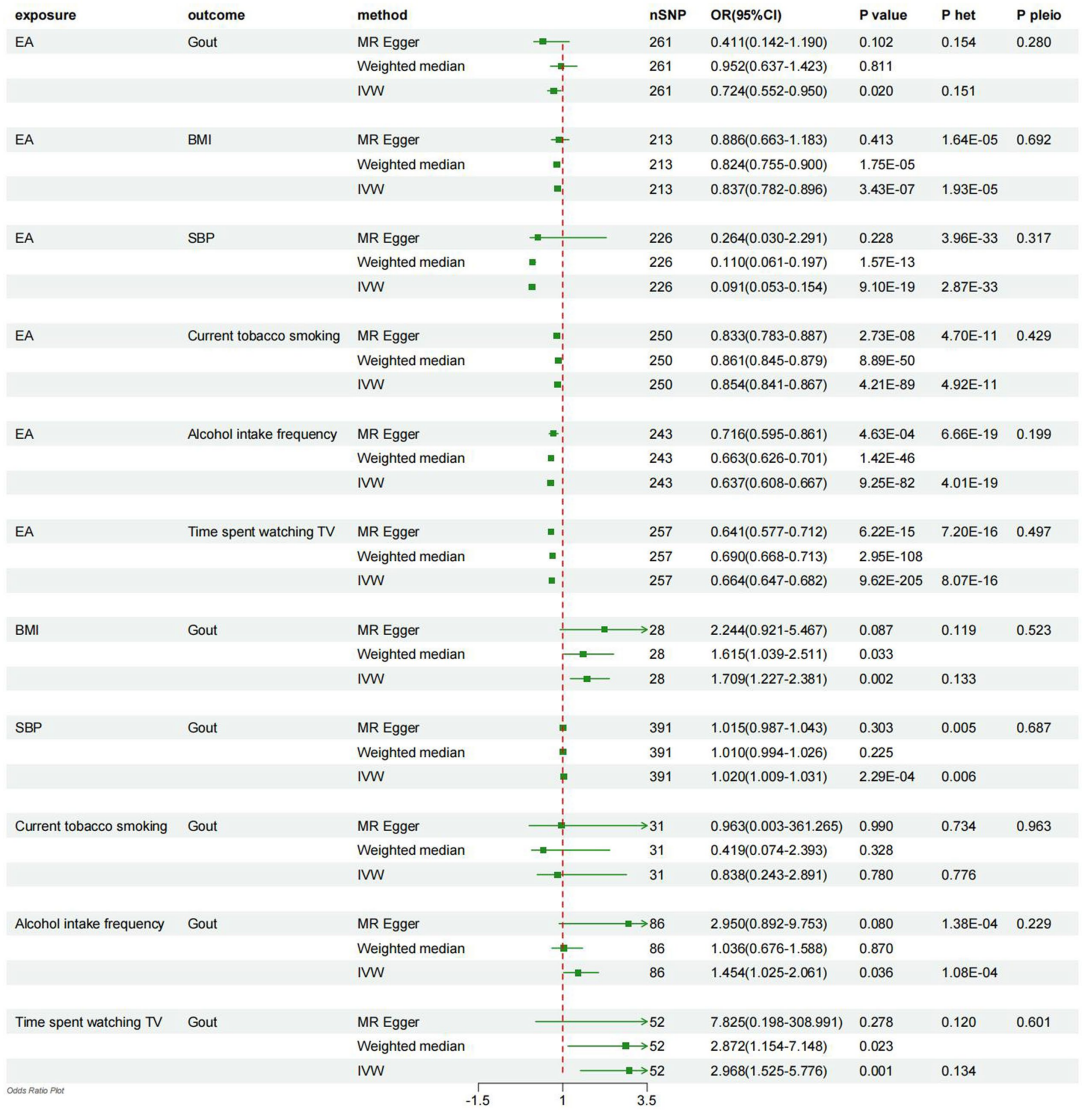
Forest plot displays the results of univariable MR analyses. MR, Mendelian randomization; het, heterogeneity; pleio, pleiotropy; IVW, inverse-variance weighted; EA, education attainment; BMI, body mass index; SBP, systolic blood pressure.

According to the primary IVW method utilized, receive 4.2 years of additional education was associated with a 27.6% lower risk of gout (OR = 0.724, 95% CI = 0.552–0.950, *p* = 0.020; Figure 2). In addition, a causal relationship between EA and a decrease in BMI, SBP and LH(smoking, drinking and TV time)was established (EA – BMI: OR = 0.837, 95% CI = 0.782–0.896, *p* = 3.43E-07; EA – SBP: OR = 0.091, 95% CI = 0.053–0.154, *p* = 9. 10E-19; EA – smoking: OR = 0.854, 95% CI = 0.841–0.867, *p* = 4.21E-89; EA – drinking: OR = 0.637, 95% CI = 0.608–0.667, *p* = 9.25E-82; EA-TV time: OR = 0.664, 95% CI = 0.664–0.682, *p* = 9.62E-205; Figure 2).

### 3.2 Overall causal effects of BMI, SBP, and LH on gout

Utilizing the IVW method, it was observed that higher BMI, SBP, drinking, and longer TV time were causally associated with an increased risk of developing gout (BMI-Gout: OR = 1.709, 95% CI = 1.227–2.381, *p* = 0.002; SBP-Gout: OR = 1.020, 95% CI = 1.009–1.031, *p* = 2.29E-04; driking-Gout: OR = 1.454, 95% CI = 1.025–2.061, *p* = 0.036; TV time-Gout: OR = 2.968, 95% CI = 1.525–5.776, *p* = 0.001; Figure 2). However, the analysis did not reveal a causal relationship between smoking and gout (smoking-Gout: OR = 0.838, 95% CI = 0.243–2.891, *p* = 0.780; Figure 2).

### 3.3 Mediating effect of BMI, SBP and LH on EA and gout

Although a causal relationship between smoking and gout was not observed, it was identified that education level had a causal impact on BMI, SBP, drinking, and TV time, and these factors also demonstrated causal effects on gout risk. Therefore, it was hypothesized that, excluding smoking, these four IPS and LH factors served as mediators in the connection between education level and gout risk. Subsequently, further analyses were conducted to evaluate the mediating effect of these four factors on the association between EA and gout risk.

**Figure 3:**
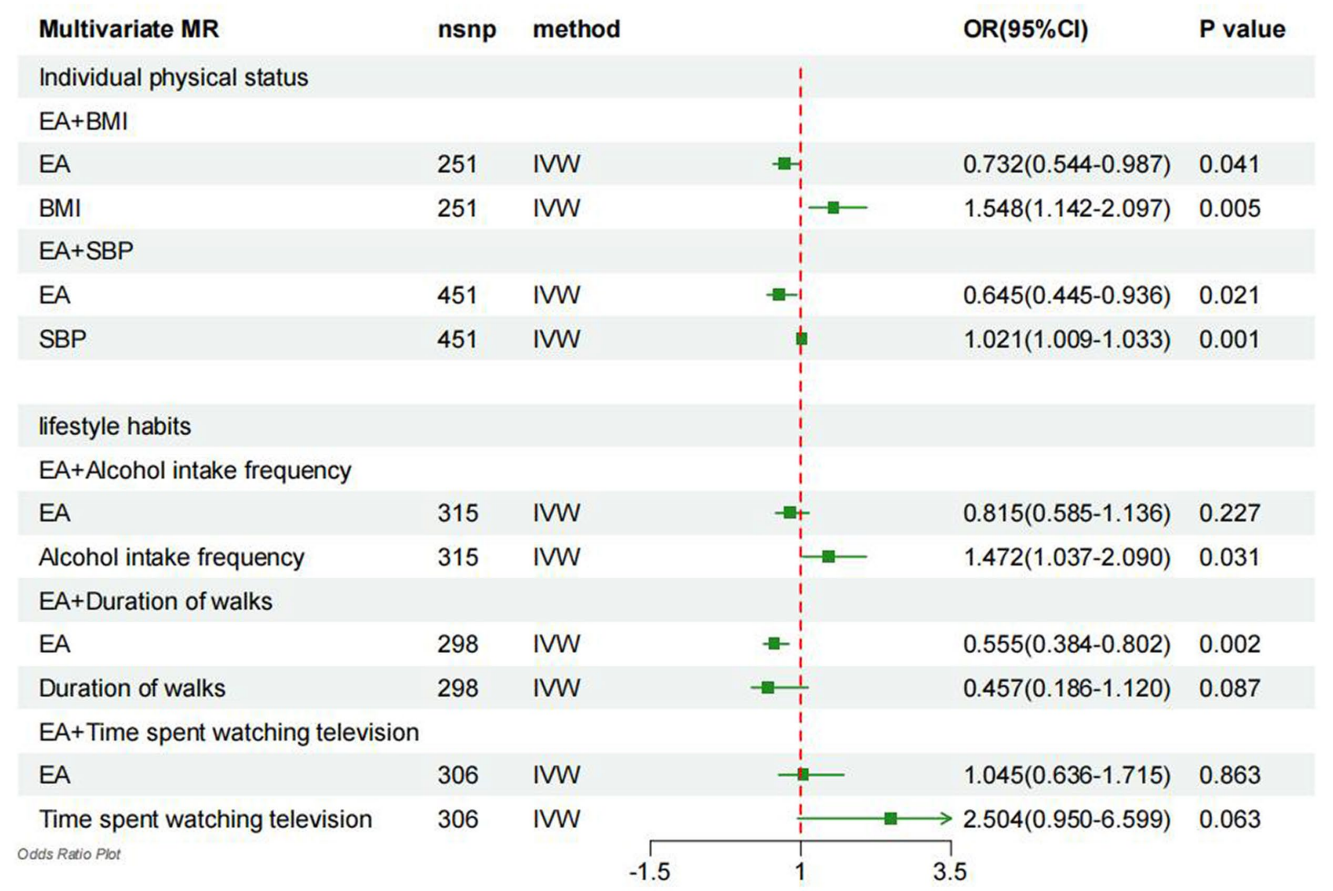
Forest plot denotes the results of multivariable MR analyses. MR, Mendelian randomization; IVW, inverse-variance weighted; EA, education attainment; BMI, body mass index; SBP, systolic blood pressure.

After adjusting for EA, it was observed that higher BMI was linked to a 54.8% increase in the risk of gout (OR = 1.548, 95% CI = 1.142–2.097, *p* = 0.005; Figure 3). Therefore, the mediating effect of BMI on the causal relationship between EA and gout risk was estimated at 27.17%. Moreover, after adjusting for EA, higher SBP was associated with a 2.1% increase in gout risk (OR = 1.021, 95% CI = 1.009–1.033, *p* = 0.001; Figure 3), suggesting that SBP mediated 14.83% of the causal effect between EA and gout risk. Additionally, adjusting for EA revealed that higher drinking led to a 47.2% increase in gout risk (OR = 1.472, 95% CI = 1.037–2.090, *p* = 0.031; Figure 3), indicating that drinking mediated 51.33% of the causal effect between EA and gout risk. Furthermore, after adjusting for EA, TV time was associated with a 150.5% increase in gout risk (OR = 2.504, 95% CI = 0.950–6.599, *p* = 0.063; Figure 3), signifying that TV time mediated 1.10% of the causal effect between EA and gout risk.

### 3.4 Sensitivity analysis

Finally, sensitivity analyses were carried out employing weighted median regression and MR-Egger methods, yielding estimates with analogous trends but diminished confidence levels (Figure 2). The heterogeneity among the included SNPs was assessed through the utilization of Cochran’s Q statistic within the IVW and MR-Egger models. The findings revealed the presence of potential heterogeneity in the majority of analyses (Figure 2). Nonetheless, it is noteworthy that this phenomenon was deemed acceptable, given that the principal outcomes were predicated upon the utilization of the random-effects IVW methodology (30). Additionally, MR-Egger regression intercepts did not indicate horizontal pleiotropy, suggesting that the current MR analysis was not influenced by such bias (*p* > 0.05; Figure 2).

## 4 Discussion

Results of our two-sample MR study suggest that to receive 4.2 years of additional education is associated with a reduced risk of gout. In addition, our rigorous MVMR and mediation analysis revealed the interesting role of elevated BMI, increased SBP, increased drinking, and extended TV time as mediating factors, depicting causal trajectories from EA to gout manifestations. This nuanced understanding has profound implications for constructing a mechanistic blueprint linking behavioral and metabolic risk factors to gout, effectively guiding prevention and treatment efforts against this urate crystal-driven inflammatory arthritis. Of these clear causal relationships, drinking appears to be the most important, accounting for an estimated 51.33% of the total effect of EA on gout. In contrast, BMI mediated 27.17%, SBP 14.83%, and TV time mediated 1.10%. Interestingly, our study did not consider smoking as a contributing factor to the development of gout due to differences in educational levels.

Inequality in EA remains a persistent social challenge implicitly linked to socio-economic and individual poverty (31). Historically, studies have shown that poor educational achievement is a potential catalyst for gout attacks (9, 10). Our survey is consistent with this view, documenting a clear negative correlation between EA and gout prevalence. Outstanding educational achievements often equip individuals with the awareness and improved living conditions necessary for a healthy lifestyle, thus decisively preventing the manifestations of gout (32–34). In addition, for those patients with gout, higher education can improve their ability to comply with health care guidelines and manage their personal life environment, thereby suppressing gout recurrence (35, 36). A revealing meta-analysis showed that of all inflammatory arthritis diseases, patients with gout had the highest rate of treatment non-adherence, reaching a staggering 90% (37). Another study conducted by Gisele Zandman-Goddard et al. revealed that of the 7,644 gout patients surveyed in Israel, only one in six adhered to allopurinol treatment (38). Higher levels of education may be an effective lever to improve medical adherence, which in turn improves the quality of life of people with gout (39).

Existing research suggests that the manifestation of gout is closely related to an individual’s physical health parameters, such as high blood pressure and obesity, which in turn are related to an individual’s EA (12, 40–43). The results of a previous survey suggest that people with high blood pressure have a two-to three-fold higher risk of developing gout (44). Elevated SBP has been shown in many studies to be a predictor of hyperuricaemia and careful SBP management has a positive effect on reducing the incidence of gout (45–47). Natalie McCormick et al. described a causal relationship between obesity and gout in a focused cohort study. The hypothesis is that without targeted weight loss, the risk of gout in obese men persists, even with other mitigation measures such as abstinence from alcohol, adherence to high DASH scores, and avoidance of diuretics (48). Our study reinforces the assertions of these previous explorations. Through MVMR analysis, this study determined that SBP and BMI mediated the occurrence of gout due to low EA by 14.83 and 27.17%, respectively. These findings suggest that lowering BMI and preventing hypertensive disorders can be effective strategies to stop gout attacks, especially in less educated people. Therefore, maintaining optimal IPS, such as proper weight range and normal blood pressure, plays an important role in preventing gout.

Previous surveys have consistently highlighted the strong link between EA and LH, including drinking, smoking and physical activity (32, 49, 50). These factors are inevitably intertwined with the onset of gout (51–53). Recent analysis by Lin Han et al. To determine that chronic and excessive alcohol consumption is a key predictor of tophi and subcutaneous tophi manifestations in patients with gout. In addition, their report linked weekly alcohol consumption to tophi progression in individuals who already had speculative tophi (54). These findings were corroborated by a study conducted by Tuhina Neogi et al., which demonstrated that the risk of gout recurrence was elevated irrespective of the type of alcoholic beverage consumed, and was linked to occasional alcohol intake (55). Consistent with these observations, our findings suggest that drinking is associated with EA and has an impact on gout prevalence—a relationship estimated to account for 51.33% of the genetic factor’s influence on disease progression. Similarly, physical activity is becoming an important mechanism for preventing gout (34). Increased TV time means less time spent on physical health, and our calculations show that TV time contributes 1.10% to education-mediated gout progression. These findings strongly suggest that adopting a healthier LH – based on curbing excessive alcohol consumption and promoting physical activity – constitutes an important prevention strategy to reduce the incidence of gout in people with lower levels of education.

Over time, the so-called causal relationship between smoking and gout remains a hotly debated topic (56). Various observational studies have come to different conclusions; While some believe that smoking may reduce the incidence of gout, others categorically deny that smoking has any substantial effect on gout (14, 51, 57). Early two-way Mendelian randomization studies confirmed the lack of a causal relationship between smoking and gout, adding weight to the latter position (58). Our empirical evidence echoes these conclusions, and our data show that smoking does not mediate the relationship between educational attainment and gout prevalence. Therefore, it is reasonable to assume that smoking has no effect on preventing gout.

Our survey, while illuminating, also acknowledges certain limitations. Initially, despite the robustness of our MR study, this research could not completely rule out the possibility of horizontal pleiotropy or other direct causal pathways. Nevertheless, the authenticity of our method is supported by interceptions from our validated MR-Egger regression analysis, indicating that there is no evidence of universal horizontal pleiotropy. Second, the GWAS database this study used primarily categorizes individuals of European ancestry, which may inevitably limit the broad applicability of our findings to populations of different ancestry. This inherent bias may have implications for the generalizability of our findings. Third, while this study utilized multivariate MR to examine the mediators and their respective mediating proportions of educational attainment inequality on gout occurrence, it is important to note that these mediating proportions may interact with one another, and the joint proportions after accounting for this interaction have yet to be quantified.

## 5 Conclusion

This study provides evidence of a causal relationship between lower levels of education and increased rates of gout. Drinking accounted for a large proportion of this effect, regulating 51.33% of the association. BMI became another important mediating variable, explaining 27.17% of the total effect. In addition, SBP plays a significant mediating role, accounting for 14.83%. Therefore, for individuals with limited access to educational resources, adopting strategies such as moderating alcohol intake and incorporating regular exercise into their routine to maintain a healthy body mass index and blood pressure may prove effective in preventing gout.

## Data availability statement

The original contributions presented in the study are included in the article/Supplementary material, further inquiries can be directed to the corresponding author.

## Ethics statement

Ethical approval was not required for the studies involving humans because the ethical review and approval for the original studies are available in the respective publications. Informed consent was obtained from all participants involved in the original genome-wide association studies. The studies were conducted in accordance with the local legislation and institutional requirements. The participants provided their written informed consent to participate in this study.

## Author contributions

XH: Project administration, Writing – review & editing, Conceptualization, Formal Analysis, Methodology, Resources, Software, Visualization, Writing – original draft. XC: Resources, Software, Visualization, Writing – original draft, Data curation, Formal analysis, Methodology. QL: Funding acquisition, Project administration, Resources, Supervision, Validation, Writing – review & editing. ZZ: Formal analysis, Investigation, Methodology, Resources, Software, Writing – original draft. JM: Resources, Supervision, Validation, Writing – review & editing. YL: Resources, Supervision, Validation, Writing – review & editing. JW: Funding acquisition, Project administration, Resources, Supervision, Writing – review & editing.

## Footnotes

^1^ https://www.finngen.fi/en

^2^ https://gwas.mrcieu.ac.uk/datasets/

^3^ http://www.nealelab.is/

## Glossary

EA: education attainment
GWAS: genome-wide association studies
SBP: systolic blood pressure
IVs: instrumental variables
IVW: inverse-variance weighted
MR: Mendelian randomization
OR: odds ratio
SD: standard deviation
SNPs: single-nucleotide polymorphisms
BMI: body mass index
SSGAC: Social Science Genetic Association Consortium
MRC-IEU: Medical Research Council Integrative Epidemiology Unit
MVMR: multivariate Mendelian randomization
TV: television
IPS: individual physical status
LH: lifestyle habits
MR-PRESSO: MR-Pleiotropy Residual Sum and Outlier
