# Supplementary material for: The relationship between education attainment and gout, and the mediating role of modifiable risk factors: a Mendelian randomization study
Source: Front Public Health. 2024 Jan 8;11:1269426. doi: 10.3389/fpubh.2023.1269426 (PMC10800502; doi:10.3389/fpubh.2023.1269426)
Supplement: Supplementary file 1 [file Table_1.DOCX]

**Table 1** Details of datasets included in analyses

|  | Trait | Sample size (Case/Control) | Population | Consortium | Reference | GWAS-ID | SNPs | Year |
| --- | --- | --- | --- | --- | --- | --- | --- | --- |
| Exposures | Education attainment | 766,345 | European | SSGAC | James J Lee et al. | ieu-a-1239 | 10,101,242 | 2018 |
| Outcome | Gout | 3,576/ 147,221 | European | Finngen | https://www.finngen.fi/en | finn-b-M13_GOUT | 16,380,152 | 2021 |
| Mediators | BMI | 152,893/ 2,477,659 | European | GIANT | Adam E Locke et al. | ieu-a-785 | 2,477,659 | 2015 |
|  | SBP | 757,601 | European | ICBP | Evangelos Evangelou et al. | ieu-b-38 | 7,088,083 | 2018 |
|  | Current tobacco smoking | 462,434 | European | MRC-IEU | Gibran Hemani et al. | ukb-b-223 | 9,851,867 | 2018 |
|  | Alcohol intake frequency | 462,346 | European | MRC-IEU | Gibran Hemani et al. | ukb-b-5779 | 9,851,867 | 2018 |
|  | Time spent watching television | 319,740 | European | Neale Lab | Neale et al. | ukb-a-5 | 10,894,596 | 2017 |

Abbreviation: SSGAC: Social Science Genetic Association Consortium; BMI: Body Mass Index; GIANT: Genetic Investigation of ANthropometric Traits; SBP: Systolic Blood Pressure; ICBP: International Consortium of Blood Pressure; MRC-IEU: Medical Research Council Integrative Epidemiology Unit.
